# Survival and quality of life after surgical aortic valve replacement in octogenarians

**DOI:** 10.1186/s13019-016-0432-0

**Published:** 2016-03-19

**Authors:** Wouter W. Jansen Klomp, Arno P. Nierich, Linda M. Peelen, George J. Brandon Bravo Bruinsma, Jan-Henk E. Dambrink, Karel G. M. Moons, Arnoud W. J. van’t Hof

**Affiliations:** Department of Cardiology, Isala V2.2, Isala Clinics, Dokter van Heesweg 2, 8025AB Zwolle, The Netherlands; Department of Clinical Epidemiology, Julius Center for Health Sciences and Primary Care, University Medical Center Utrecht, Universiteitsweg 100, Utrecht, 3584CG The Netherlands; Anaesthesiology and Intensive care, Isala Clinics, Dokter van Heesweg 2, 8025AB Zwolle, The Netherlands; Cardiothoracic Surgery, Isala Clinics, Dokter van Heesweg 2, 8025AB Zwolle, The Netherlands

**Keywords:** Aortic Valve Replacement, Aortic Valve Stenosis, Cardiac Surgery, Elderly, Octogenarian, Quality of Life

## Abstract

**Background:**

In patients with symptomatic severe aortic stenosis, advanced age is often a reason for a transcatheter rather than surgical aortic valve replacement. In this pre-transcathter cohort we had the unique oportunity to study outcomes after surgical aortic valve replacement for severe aortic stenosis in patients who might currently be triaged to a percutaneous approach.

**Methods:**

In a prospective single-center cohort study we compared the incidence of peri-operative complications, mortality, and health-related quality of life in octogenarians versus patients aged <80 years. The quality of life was measured using the SF-36 questionnaire and expressed as a physical and mental component score (PCS and MCS respectively); a score of 50 equals the average score in the age-matched general population. The association between age and the component scores at one-year follow-up was studied with the use of linear regression, corrected for a set of confounding variables.

**Results:**

We included 762 patients, of whom 21.4 % was aged >80 and 49.0 % underwent concomitant revascularization. In octogenarians, the incidence of post-operative delirium was 11.0 %, which was higher than in patients aged below 80 (6.2 %, *p* = 0.034); the operative mortality (1.9 % vs. 2.9 %; *p* = 0.59) and long-term survival were not different however (log-rank *p* = 0.75). In octogenarians, the quality of life was impaired 30-days after surgery (PCS 45.01, *p* <0.001; MCS 48.21, *p* = 0.04), which improved towards or above normal values at one-year follow-up (PCS: 49.92, *p* = 0.67, MCS: 52.55, *p* < 0.001). After correction for confounding, age was not significantly associated with the one-year PCS (β 0.08 per year, *p* = 0.34) or MCS (β 0.08 per year, *p* = 0.32).

**Conclusions:**

This pre-transcatheter study showed that surgical aortic valve replacement in octogenarians could be performed with very low mortality, and with a relevant and significant increase of the quality of life towards normal values. Also, age was not associated with a lower PCS or MCS one-year after surgery.

**Electronic supplementary material:**

The online version of this article (doi:10.1186/s13019-016-0432-0) contains supplementary material, which is available to authorized users.

## Background

Surgical aortic valve replacement (AVR) has long been the only effective treatment for symptomatic severe aortic stenosis (AS) [1–3]. The introduction of catheter-based techniques questioned existing treatment strategies for patients with AS. One obvious, but difficult challenge is to assess in which patients transcatheter aortic valve replacement (TAVR) is preferable, and in whom a surgical intervention remains the treatment of choice. Based on the results of four randomized trials [4–7] and multiple registries, current guidelines recommend TAVR in patients who are either not suitable for surgery or have a very high surgical risk [2, 8]. These recommendations leave room for interpretation and in daily clinical practice advanced age alone is often the reason to prefer a percutaneous approach [9]. This may indicate that physicians expect a poorer outcome after surgical AVR in elderly patients.

In this study we investigate data from a large cohort of surgical AVR patients that underwent surgery before TAVR became available in our clinic. We will hence not directly compare TAVR with surgical AVR, but we have the unique opportunity to assess outcomes of surgical AVR in patients who may nowadays be triaged to a percutaneous approach.

The aim of our study was to investigate the influence of age on postoperative outcomes, in particular comparing octogenarians to younger patients. Additional to post-operative complications and long-term mortality we also studied the health-related quality of life (HRQoL) after one year, an outcome increasingly recognized as an outcome of importance [2], and its change from baseline HRQoL for patients of different age groups.

## Methods

In this prospective, observational cohort study we included consecutive patients with symptomatic AS who underwent elective surgery for aortic stenosis, either isolated AVR or AVR with concomitant coronary artery bypass grafting (CABG). Patients were operated in a single institution (Isala clinics, Zwolle, The Netherlands) between November 1^st^ 2007 and August 1^st^ 2011. During this period, the institution did not perform percutaneous procedures yet; therefore, patients who would currently be considered for a percutaneous approach were part in this surgical cohort.

Patients gave written informed consent for the systematic collection of data in an ongoing registry of cardiothoracic surgery in our institution. We excluded patients who refused informed consent, who had a non-stenotic valve, active endocarditis or a previous AVR during the study period from our analysis. The institution’s ethical committee approved the protocol (reference: 12.0881n) and waived the need for formal evaluation according to the Dutch Law on Scientific Medical Research with Humans. Data were anonymized before data analysis, and the study conforms to the principles outlined in the Helsinki declaration.

### Data collection and end-points

Data were collected in an ongoing registry, which included baseline characteristics, perioperative data, in-hospital outcomes, and follow-up for clinical events and quality of life until one year after surgery. The aortic peak gradient was retrospectively registered through a review of electronic charts. End-points were reported according to the “Valve Academic Research Consortium-2 (VARC-2) criteria [10]. In-hospital stroke was diagnosed by an attending neurologist, and usually supported by cerebral computed tomography imaging. Presence and severity of postoperative kidney injury were defined according to the Acute Kidney Injury Network (AKIN) classification [11]. Delirium was assessed using the delirium observation screening (DOS) scale, which was performed daily until hospital discharge. Operative mortality was defined as 30-day mortality or in-hospital mortality if postoperative hospital stay exceeded 30 days. Long-term all-cause mortality was extracted from the Dutch Municipal Personal Records Database on April 6^th^, 2012.

The HRQoL was measured with the Medical Outcomes Study Short-Form 36 (SF-36), a widely used and well-validated self-administered instrument [12, 13]. Patients completed the test preoperatively, and at 30-days and one year after intervention. According to the guidelines for this instrument the 36 questions were subsequently grouped into eight health scales (physical functioning, role-physical, bodily pain, general health, vitality, social functioning, role-emotional, and mental health) and two summary measures (the physical component score [PCS] and mental component score [MCS]).

### Statistical analysis

Throughout the analyses we compared octogenarians (defined as an age ≥80 years) with patients aged less than 80 years. The baseline characteristics and in-hospital outcomes are presented as frequencies and percentages of total for dichotomous variables, as the mean and standard deviation (SD) for normally distributed data or the median and interquartile range (IQR) for non-normally distributed data respectively. Differences in categorical variables were assessed as appropriate with a chi-square test or a Fisher’s exact test. Differences in continuous variables were tested using a Students’ *t*-test or Mann–Whitney-*U* test. Survival plots were calculated with Kaplan-Meier (KM) statistics, as a reference we also depicted the predicted survival of age- and sex matched Dutch inhabitants reported by Statistics Netherlands (www.cbs.nl). Differences in KM-estimated survival were tested with a log-rank test.

The PCS and MCS were normalized such that a score of 50 (standard deviation [SD] 10) equalled the mean score of an age-matched Dutch reference population [13]. We calculated the difference between the summary scores at baseline versus the scores at 30-days and one year follow-up, and tested these differences with a paired *t*-test. Also, we tested for a difference in the change in summary scores in octogenarians compared to younger patients using an independent samples *t*-test, which was corrected for the baseline scores.

To study the clinical relevance of a difference (rather than only statistical significance) we also calculated Cohen’s effect size by dividing the difference between two measurements through the SD at baseline. Furthermore, we calculated the percentage of patients in whom a minimal clinically important difference (MCID) was observed, defined as a difference ≥2.5 points above or below the baseline score [14].

Finally, we studied the influence of age on the difference in the component scores at one-year follow-up compared to baseline. The respective scores of the PCS and MCS were included as the dependent variable in a univariable generalized linear regression model (crude association), and were then corrected for several predefined covariables (see Additional file 1). Sex was included in the multivariable model to study gender specific changes. Furthermore, the baseline component scores were also included, since patients with higher baseline scores generally improve less on follow-up measurements than patients with lower baseline scores [15].

Missing data were imputed since data are usually not missing completely at random, but rather selectively [16, 17]. Discarding patients with missing data may lead to biased results and furthermore a loss in precision [16, 17]. Imputation was performed in SPSS using five imputed datasets, and then pooled using Rubin’s rule (see Additional file 1 for imputation model). Throughout the analysis a level of significance of 0.05 was used. Analyses were performed in SPSS Statistics version 21.0.0 and R version 2.13.1.

## Results

### Characteristics of the patients

**Fig. 1:**
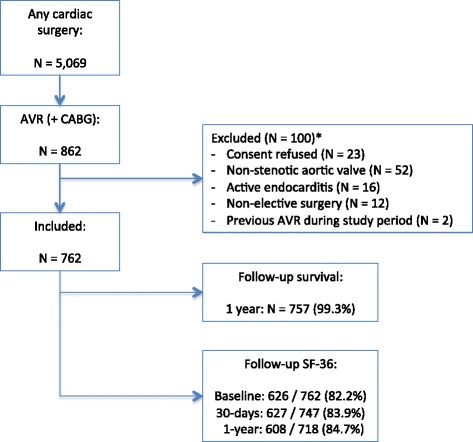
Study flowchart and follow-up. Legend: Patients were included between November 1st 2007 and August 1st 2011*One hundred patients were excluded in total; five patients met two exclusion criteria, i.e. active endocarditis and non-elective surgery. AVR = aortic valve replacement, CABG = coronary artery bypass grafting, FU = follow-up

During the inclusion period, 5,069 cardiothoracic operations were performed, of which 862 were isolated AVR or AVR with concomitant CABG. Twenty-three patients were excluded because they refused consent for the registration; another 77 patients met one or more of the exclusion criteria (Fig. 1). The final study population thus comprised 762 patients. Follow-up of the vital status was completed in 757 (99.3 %) of patients, with a median follow-up of 2.4 years (IQR 1.0–3.3). Follow-up of the SF-36 was completed by 82.2 % of patients at baseline, by 83.9 % at 30-days follow-up and 84.7 % of patients at one-year follow-up. The baseline characteristics of patients with and without missing one-year follow-up of the SF-36 questionnaire are shown in Additional file 2.

One-hundred and sixty-three patients (21.4 %) were aged 80 years or over at the date of surgery; median age of this group was 82 years, with a female predominance of 52.1 %. Octogenarians had a lower systolic left ventricular function than younger patients (*p* = 0.044), other baseline characteristics were not significantly different (Table 1). A bioprosthesis was implanted more often in octogenarians than in non-octogenarians (97.6 % vs. 90.6 %, *p* <0.001); the total cross-clamp time was shorter (82 vs. 91 min, *p* = 0.004), and combined surgery with CABG occurred in almost half of the procedures in both age groups less frequently (46.0 % vs. 49.9 %, *p* = 0.426). The EuroSCORE I predicted risk of operative mortality was higher in octogenarians than in patients aged below 80 years (10.7 % vs. 4.9 %, *p* < 0.001).

**Table 1:** Baseline characteristics and operative characteristics

		Age < 80	Age ≥ 80	
		*N* = 597	*N* = 163	*P*
Male sex		363 (60.8)	85 (52.1)	0.057
Age, years		71 (66–75)	82 (81–83)	<0.001
History of:				
DM		145 (24.4)	37 (22.7)	0.727
Hypertension		325 (54.4)	98 (60.1)	0.228
COPD		116 (19.4)	30 (18.4)	0.855
Peripheral VD		70 (11.7)	17 (10.4)	0.748
Stroke		66 (11.1)	20 (12.3)	0.768
MI		74 (12.4)	21 (12.9)	0.979
PCI		65 (10.9)	18 (11.0)	1.000
Cardiac surgery		42 (7.0)	8 (4.9)	0.428
Creatinine (mmol/L)		88 (75–104)	92 (77–110)	0.001
EuroSCORE		6 (5–7)	8 (8–10)	<0.001
Log EuroSCORE		4.9 (3.1–7.6)	10.7 (8.4–15.7)	<0.001
LVEF	>50 %	468 (78.7)	113 (69.3)	0.044
	30–50 %	103 (17.3)	40 (24.5)	
	<30 %	24 (4.0)	10 (6.1)	
NYHA	Class 1	145 (24.3)	45 (27.6)	0.149
	Class 2	342 (57.4)	80 (49.1)	
	Class 3	109 (18.3)	38 (23.3)	
Peak gradient AV (mmHg)		73 (60–90)	71 (58–88)	0.55
Operative characteristics				
Bioprosthesis		541 (90.6)	159 (97.6)	<0.001
CABG		298 (49.9)	75 (46.0)	0.426
X-clamp time (min)		91 (75–111)	82 (68–107)	0.004

Baseline chararcteristics and operative characteristics in octogenarians and patients aged less than 80 years. Values are number (%), continuous variables are presented as median (25th–75th percentile)

*DM* diabetes mellitus, *COPD* chronic obstructive pulmonary disease, *VD* vascular disease, *MI* myocardial infarction, *PCI* percutanaeous coronary intervention, *LVEF* left ventricular ejection fraction, *NYHA* New York Heart Association, *AV* aortic valve, peak gradient = maximum gradient over the aortic valve, *CABG* coronary artery bypass grafting

### In-hospital outcomes and mortality

**Fig. 2:**
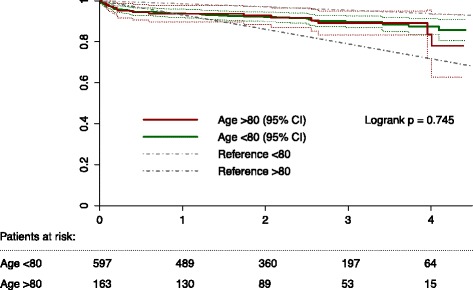
Long-term survival after aortic valve replacement. Legend: Kaplan-Meier estimated survival in octogenarians (red line and 95 % confidence interval [CI]) compared to patients aged <80 years (green line and 95 % CI); as a reference the dotted black lines depict the predicted survival for Dutch inhabitants with a similar distribution of age and sex as the two study groups

During hospitalization, octogenarians more frequently developed a delirium (11.0 % vs. 6.2 %, *p* = 0.034), and total hospital-stay was longer (median (IQR) of 7 (5–9) and 7 (5–12) respectively, *p* = 0.031; Table 2). The incidence of stroke (3.1 % vs. 3.5 %, *p* = 0.97), myocardial infarction (2.6 % vs. 3.0 %, *p* = 1.00) and acute kidney injury (9.2 % vs. 7.6 %, *p* = 0.22) did not differ significantly between the two groups. Two octogenarians died before hospital discharge, one additional patient died within 30-days of surgery, thus the operative mortality was 1.9 %, compared to 2.9 % in patients aged <80 (*p* = 0.59). Both one-year mortality (6.5 % vs. 6.3 %, *p* = 1.00) and long-term mortality were similar for octogenarians and patients aged < 80 years (log-rank *p* = 0.745; Fig. 2).

**Table 2:** Patient outcomes

		Age < 80	Age ≥ 80	
		*N* = 597	*N* = 163	*P*
In-hospital Outcomes				
Delirium		37 (6.2)	18 (11.0)	0.034
Stroke		21 (3.5)	5 (3.1)	0.97
MI		17 (3.0)	4 (2.6)	1.00^a^
AKIN	No injury	537 (92.4)	148 (90.8)	0.22
	Stage 1	36 (6.2)	12 (7.4)	
	Stage 2	5 (0.9)	0 (0)	
	Stage 3	3 (0.5)	3 (1.8)	
Hospital stay (days)		7 (5–9)	7 (5–12)	0.031
Mortality				
In-hospital		13 (2.2)	2 (1.2)	0.75^a^
Operative		17 (2.9)	3 (1.9)	0.59^a^
1-Year		33 (6.3)	9 (6.5)	1.00

Comparison of operative chararcteristics and patient outcomes. Operative mortality is either 30-day or in-hospital mortality

*MI* myocardial infarction, *AKIN* acute kidney injury network

^a^ Fisher’s Exact test

**Table 3:** Health-related quality of life in patients undergoing surgical AVR

	Baseline	30 days					1 year				
	Score	Score	Change from baseline^a^	P for change	Cohen’s effect size^b^	% reaching MCID	Score	Change from baseline^a^	P for change	Cohen’s effect size^2^	% reaching MCID
PCS											
Age < 80	44.27	44.11	−0.16	0.73	−0.02	39.0	51.34	6.95	<0.001	0.78	66.8
Age > 80	44.86	45.01	0.09†	0.93	0.01	40.1	49.92	4.84†	<0.001	0.54	60.7
MCS											
Age < 80	49.12	48.33	−0.81	0.073	−0.52	36.2	50.83	1.67	0.006	0.22	44.2
Age > 80	51.17	48.21	−2.96†	0.002	−0.64	32.5	52.55	1.31†	0.126	0.14	39.6

^a^The “change from baseline” in octogenarians was compared to patients aged <80 years using an independent samples *t* test and corrected for the baseline component score, where † indicates *p* > 0.05

^b^An effect size of <0.20 can be considered as clinically irrelevant, 0.20–0.49 as small, 0.50–0.79 as moderate and > 0.80 as large. PCS = physical component score, MCS = mental component score, MCID = minimal clinically important difference

### Health-related quality of life

The PCS in octogenarians was 44.68 at baseline, which was lower than the average score of 50 in the age-matched reference population (*p* <0.001; Table 3). The PCS remained impaired 30-days after surgery (45.01, *p* <0.001) but improved towards a normal level one year after surgery (49.92, *p* = 0.67). The one-year PCS in octogenarians was also comparable to patients aged below 80 (49.92 vs. 51.34, *p* = 0.12), but the increase from baseline was smaller in octogenarians (4.84 vs. 6.95, *p* = 0.04), as was Cohen’s D (0.54 vs. 0.78, *p* = 0.04).

The MCS in octogenarians was 51.17 at baseline, which was higher than the average score in the reference population (*p* = 0.04). Thirty-days after surgery the MCS had reduced towards a below-average score (48.21, *p* = 0.04), but at one-year follow-up the MCS was again above expected (52.55, *p* < 0.001). The MCS one year after surgery was also higher in octogenarians compared to patients aged below 80 years (MCS 52.55 vs. 50.83, *p* < 0.001); however, the increase from baseline was similar (1.31 vs. 1.67, *p* = 0.38) and Cohen’s effect size was lower (0.14 vs. 0.22, *p* = 0.01).

### Association between age and HRQoL

Increasing age was associated with a higher PCS at 30-days follow-up, both in a univariable model (β 0.20 per year, 95 % CI: 0.14–0.27, *p* <0.001) and when corrected for baseline characteristics (β 0.30, 95 % CI: 0.08–0.15, *p* <0.001; Table 4). One year after surgery age was however not associated with the PCS (multivariable model: β 0.08, 95 % CI: −0.11–0.16, *p* = 0.34). Also, age was not associated with the MCS at 30-days follow-up (β -0.07, 95 % CI: −0.22–0.83, *p* = 0.37), or 1-year after surgery (β 0.08, 95 % CI: −0.08–0.25, *p* = 0.32).

**Table 4:** Association between age and the PCS and MCS scores at 30 days and 1 year follow-up

	PCS – 30 days			PCS – 1 year		
	β	(95 % CI)	*P*	β	(95 % CI)	*P*
Age	0.20	(0.14–0.27)	<0.001	−0.00	(−0.11–0.10)	0.90
Age (+ baseline PCS)	0.16	(0.10–0.22)	<0.001	−0.07	(−0.17–0.04)	0.19
Age (full model)	0.30	(0.08–0.15)	<0.001	0.08	(−0.11–0.16)	0.34
	MCS – 30 days			MCS – 1 year		
	β	(95 % CI)	*P*	β	(95 % CI)	*P*
Age	−0.05	(−0.14–0.03)	0.22	0.07	(−0.02–0.15)	0.11
Age (+ baseline)	−0.08	(−0.16–0.00)	0.05	0.05	(−0.03–0.14)	0.20
Age (full model)	−0.07	(−0.22–0.83)	0.37	0.08	(−0.08–0.25)	0.32

Table shows the univariable and multivariably adjusted asociation between age and the component scores; the full model also included: sex, chronic obstructive pulmonary disease, diabetes mellitus, extracardiac atherosclerosis, history of stroke, history of myocardial infarction, previous percutaneous coronary intervention, previous cardic surgery, NYHA class, CCS class, left ventricular function, concomitant coronary artery bypass grafting, aortic peak gradient, logistic EuroSCORE and baseline physical component score for the PCS model, or baseline mental component score for the MCS model. PCS = physical component score, MCS = mental component score

## Discussion

This study of pre-TAVR patients showed that surgical AVR in octogenarians could be performed with very low postoperative mortality, and a relevant and significant increase of the HRQoL towards normal values. Also, age was not associated with a lower PCS or MCS one-year after surgery.

### Outcomes and survival

Post-operative delirium was more often observed in octogenarians compared to younger patients (11.0 % vs. 6.2 %, *p* = 0.03), which may reflect an increased vulnerability of the aging brain for ischemic cerebral damage. Several perioperative processes are associated with this type of injury, including the embolization of particulate emboli, hypotension and inflammation [18]. Indeed, age was previously found to be one of the most important risk-factors for post-operative delirium [19]. However, the incidence of stroke, which is regarded to be in the same spectrum of cerebral (embolic) injury as delirium, was not increased in octogenarians.

Mortality rates were low, with an observed operative mortality of 1.9 % in octogenarians and 2.9 % (*p* = 0.59) in younger patients. These rates are considerably lower than reported in previous studies, which ranged from 4.0–16 % [20–23]. One year after surgery 93.5 % of octogenarians were alive, and the long-term survival up to 4 years after surgery was similar compared to younger patients. These outcomes contrast with the grim prognosis of medically treated severe AS [1], and based on our results one may question if age alone is a valid motivation to either refrain from surgery or to prefer a percutaneous treatment. Interestingly, from one year after surgery onwards, the long-term survival of octogenarians was better than that of Dutch inhabitants of the same age and sex. Although it is well accepted that surgery for aortic stenosis improves life expectancy, the improved performance compared to the general population is likely to also indicate the selection of a healthy population in our study, which may have positively influenced the results of our study. Yet, over 20 % of patients was aged ≥80 years, which is in the higher range of other registries, and indicates a pro-active attitude towards surgical management in these patients [20, 22]. Also, we included patients with concomitant revascularization as recommended, since both survival and HRQoL are known to be reduced in these patients [24].

### Health-related quality of life

One month after surgery the PCS and MCS seemed to decrease rather than to improve, which may be related to the burdensome effects associated with sternotomy. This was also shown in the PARTNER trial, which randomly allocated patients to a surgical or percutaneous AVR. In that study, one month after intervention the HRQoL had increased in the latter, but not in the former group [25]. However, at 6 and 12 months follow-up a similar increase of the PCS was observed after both approaches. Similarly in our study, the PCS and MCS one year after surgery had increased relevantly and significantly towards a level expected for the reference population.

### Association between age and HrQoL

Age was not associated with the PCS score at thirty days and one-year follow-up. Furthermore, age was positively associated with the MCS at 30-days follow-up. Although the reasons for this positive association have to be speculated, these results indicate that older patients can expect a relative quality of life at least similar to younger patients.

As expected, a higher baseline component score was associated with a lower component score at one-year follow-up. This is probably caused by regression to the mean, as was described previously in the serial analysis of HRQoL measurements [15]. That is, patients who already have a high baseline score are less likely to have an even higher score at follow-up, while patients with a low baseline score are less likely to have an even lower score. To correct for this potential bias, we included the baseline component scales in the multivariable linear regression analyses.

### Limitations

The results of this study should be appreciated with consideration of its limitations. First, as in any observational study, the efficacy of the intervention itself could not be studied, as this would require a control group of similar patients without surgical AVR. However, as a general reference, we compared both the survival and HRQoL to a matched general population. Long-term survival in octogenarians was better than expected for age- and sex matched Dutch inhabitants, which is likely to indicate that the octogenarians selected as suitable candidates for surgery were in relative good physical health. Yet, as mentioned, the proportion of octogenarians in our study is comparable to other registries. Also, mortality was well below predicted based on the original EuroSCORE.

One-year follow-up of the SF-36 questionnaire was missing in approximately 15 % of patients. Missing follow-up status was associated a higher preoperative EuroSCORE, but this difference was non-significant within patients alive at one-year (i.e. missingness not due to mortality; Additional file 2). In such a situation where data are not “missing completely at random”, it has been recommended to multiply impute missing data [17]. In a sensitivity analysis that included patients with complete cases only, our results did not change substantially (Additional file 3).

Finally, as recommended by the VARC-2 criteria, postoperative kidney injury was defined according to the AKIN classification in which kidney injury is categorized both according to creatinine levels and urine output (whichever falls in the worse category) [10]. The latter was not recorded however, which may thus have resulted in misclassification of patients.

## Conclusion

This study of pre-TAVR patients showed that surgical AVR in octogenarians could be performed with very low mortality and a relevant and significant increase of the quality of life towards normal values. Age was not associated with a lower PCS or MCS one-year after surgery after adjustment for comorbidities. Hence, based on these results, age by itself does not serve as a contraindication for surgical AVR.

## Additional files

Additional file 1: Variables included in the multivariable and imputational models. (DOCX 89 kb) Additional file 2: Baseline characteristics of patients with missing follow-up of SF-36 data. (DOCX 88 kb) Additional file 3: SF-36 complete case analyses. (DOCX 73 kb)

## Abbreviations

AS: aortic stenosis
AVR: aortic valve replacement
CABG: concomitant coronary artery bypass grafting
DOS: delirium observation screening scale
HRQoL: health-related quality of life
IQR: interquartile range
KM: Kaplan-Meier
MCID: minimal clinically important difference
MCS: mental component score
PCS: physical component score
TAVR: transcatheter aortic valve replacement
VARC-2: valve academic research consortium-2
