# Supplementary material for: Survival and quality of life after surgical aortic valve replacement in octogenarians
Source: J Cardiothorac Surg. 2016 Mar 19;11:38. doi: 10.1186/s13019-016-0432-0 (PMC4799630; doi:10.1186/s13019-016-0432-0)
Supplement: Additional file 1: — Variables included in the multivariable and imputational models. (DOCX 89 kb) [file 13019_2016_432_MOESM1_ESM.docx]

Supplement A:

1. Variables included in the full generalized linear models:

*Sex, age, chronic obstructive pulmonary disease, diabetes mellitus, extracardiac atherosclerosis, history of stroke, history of myocardial infarction, previous percutaneous coronary intervention, previous cardic surgery, NYHA class, CCS class, left ventricular function, concomitant coronary artery bypass grafting, aortic peak gradient, logistic EuroSCORE and baseline physical component score for the PCS model, or baseline mental component score for the MCS model.*

2. Imputation model (syntax SPSS):

DATASET DECLARE Impute_AVR_QOL_SF36.

MULTIPLE IMPUTATION REOP30 LES_20 Octogenarian Logistic_ES IAVR_AVRCABG Intervention_ID PtID

Geslacht Gebdat Age_@_int Days_discharge Days_last_FU KM_status In_hospital_mortality

Thirtyday_mortality Operative_mort One_year_mort_cum TIA__30 CVA__30 KreatVoorgeschiedenis

Eerd_PTCA Eerd_cardiochir Chron_longzkte ExCard_vaatpath Zkte_neurol_dysf Instab_angina_p Hypert

DM_Cat BMI VoorgeschiedenisNierf_stoorn preop_critical_state Excorp_circ_total Aorta_occltijd_total

Bloedverl_OK Isolated_valves Beademduur Kreat_max Delier Insult Days_MC_level OpnameIC_Hours

Hosp_discharge_days SF36_VG_PF SF36_VG_RP SF36_VG_BP SF36_VG_GH SF36_VG_VT SF36_VG_SF SF36_VG_RE F36_VG_MH SF36_FU30_PF SF36_FU30_RP SF36_FU30_BP SF36_FU30_GH SF36_FU30_VT SF36_FU30_SF F36_FU30_RE SF36_FU30_MH SF36_FU1_PF SF36_FU1_RP SF36_FU1_BP SF36_FU1_GH SF36_FU1_VT SF36_FU1_SF SF36_FU1_RE SF36_FU1_MH SF36_VG_missing_2 SF36_FU30_missing_2 SF36_FU1_missing_2

/IMPUTE METHOD=AUTO NIMPUTATIONS=5 MAXPCTMISSING=NONE

/MISSINGSUMMARIES NONE

/IMPUTATIONSUMMARIES MODELS

/OUTFILE IMPUTATIONS=Impute_AVR_QOL_SF36
