# Supplementary material for: Survival and quality of life after surgical aortic valve replacement in octogenarians
Source: J Cardiothorac Surg. 2016 Mar 19;11:38. doi: 10.1186/s13019-016-0432-0 (PMC4799630; doi:10.1186/s13019-016-0432-0)
Supplement: Additional file 2: — Baseline characteristics of patients with missing follow-up of SF-36 data. (DOCX 88 kb) [file 13019_2016_432_MOESM2_ESM.docx]

|  | | One-year follow-up SF-36 | | | | | | | |  |
| --- | --- | --- | --- | --- | --- | --- | --- | --- | --- | --- |
|  | | Complete | |  | Missing  (All patients) | |  | Missing  (One-year survivors) | |  |
|  | | N = 608 | |  | N = 154 | | P* | N = 110 | | p† |
| Male sex | | 364 | (59.9) |  | 85 | (55.2) | 0.29 | 62 | (56.4) | 0.47 |
| Age, years | | 73 (67 – 78) | |  | 75 (68 – 78) | | 0.26 | 73 (67 – 78) | | 0.75 |
| History of: | |  |  |  |  |  |  |  |  |  |
| DM | | 132 | (21.8) |  | 50 | (32.7) | 0.005 | 37 | (33.9) | 0.006 |
| COPD | | 111 | (18.3) |  | 36 | (23.4) | 0.15 | 21 | (19.1) | 0.84 |
| Peripheral VD | | 66 | (10.9) |  | 21 | (13.6) | 0.33 | 13 | (11.8) | 0.77 |
| Stroke | | 71 | (11.7) |  | 16 | (10.4) | 0.65 | 7 | (6.4) | 0.10 |
| MI | | 77 | (12.7) |  | 18 | (11.7) | 0.74 | 12 | (10.9) | 0.60 |
| PCI | | 64 | (10.5) |  | 20 | (13.0) | 0.39 | 12 | (10.9) | 0.90 |
| Cardiac surgery | | 31 | (5.1) |  | 19 | (12.3) | 0.001 | 12 | (10.9) | 0.02 |
| EuroSCORE | | 6 (5 – 8) | |  | 7 (5 – 9) | | <0.001 | 7 (5 – 9) | | 0.13 |
| Log EuroSCORE | | 5.8 (3.4 – 9.5) | |  | 7.6 (4.2 – 12.8) | | <0.001 | 7.0 (3.7 – 11.2) | | 0.13 |
| LVEF | >50% | 477 | (78.8) |  | 105 | (68.2) | 0.002 | 76 | (69.1) | 0.01 |
|  | 30 – 50% | 109 | (18.0) |  | 35 | (22.7) |  | 24 | (21.8) |  |
|  | <30% | 20 | (3.3) |  | 14 | (9.1) |  | 10 | (9.1) |  |
| NYHA | Class 1 | 146 | (24.1) |  | 31 | (28.2) | 0.12 | 31 | (28.2) | 0.27 |
|  | Class 2 | 348 | (57.3) |  | 54 | (49.1) |  | 54 | (49.1) |  |
|  | Class 3 | 113 | (18.6) |  | 25 | (22.7) |  | 25 | (22.7) |  |
| Peak gradient (mmHg) | | 73 (60 – 90) | |  | 71 (56 – 90) | | 0.42 | 71 | (58 – 90) | 0.36 |

Supplement B – Baseline characteristics of patients with missing follow-up of SF-36 data.

Baseline characteristics of patients who completed the one-year follow-up of the SF-36 questionnaire, compared to * all patients with missing 1-year follow-up (i.e. including patients who died < 1 year) and † one-year survivors with missing follow-up (i.e. excluding patients who died < 1 year).
