# Supplementary material for: Survival and quality of life after surgical aortic valve replacement in octogenarians
Source: J Cardiothorac Surg. 2016 Mar 19;11:38. doi: 10.1186/s13019-016-0432-0 (PMC4799630; doi:10.1186/s13019-016-0432-0)
Supplement: Additional file 3: — SF-36 complete case analyses. (DOCX 73 kb) [file 13019_2016_432_MOESM3_ESM.docx]

Supplement C. SF-36 complete case analyses

|  |  | Baseline | 30 days | | | | | 1 year | | | | |
| --- | --- | --- | --- | --- | --- | --- | --- | --- | --- | --- | --- | --- |
|  |  | Score | Score | Change from baseline^1^ | P for change | Cohen’s effect size^2^ | % reaching MCID | Score | Change from baseline^1^ | P for change | Cohen’s effect size^2^ | % reaching MCID |
| **PCS** |  |  |  |  |  |  |  |  |  |  |  |  |
| Age < 80 |  | 45.02 | 44.48 | -0.31 | 0.521 | -0.04 | 37.7 | 51.55 | 6.58 | <0.001 | 0.74 | 65.3 |
| Age > 80 |  | 45.48 | 45.51 | -0.23† | 0.819 | -0.03 | 35.1 | 50.48 | 4.63† | <0.001 | 0.52 | 63.9 |
|  |  |  |  |  |  |  |  |  |  |  |  |  |
| **MCS** |  |  |  |  |  |  |  |  |  |  |  |  |
| Age < 80 |  | 49.50 | 49.13 | -0.38 | 0.450 | -0.51 | 34.9 | 50.97 | 1.46 | 0.005 | 0.22 | 42.1 |
| Age > 80 |  | 51.77 | 48.22 | -3.70* | 0.001 | -0.67 | 26.8 | 52.28 | 0.61† | 0.474 | -0.17 | 33.3 |

Data depict the health-related quality of life of patients with a complete follow-up of the SF-36 one year after intervention. ^1^ The “change from baseline” in octogenarians was compared to patients aged <80 years, where † indicates P = NS. ^2^An effect size of <0.20 can be considered as clinically irrelevant, 0.20-0.49 as small, 0.50-0.79 as moderate and > 0.80 as large. PCS = physical component score, MCS = mental component score, MCID = minimal clinically important difference.
